# Mitophagy in Astrocytes Is Required for the Health of Optic Nerve

**DOI:** 10.3390/cells12202496

**Published:** 2023-10-20

**Authors:** Meysam Yazdankhah, Sayan Ghosh, Haitao Liu, Stacey Hose, J. Samuel Zigler, Debasish Sinha

**Affiliations:** 1 Department of Ophthalmology, University of Pittsburgh School of Medicine, Pittsburgh, PA 15213, USA; sayang@pitt.edu (S.G.); hal140@pitt.edu (H.L.); stacey.hose@pitt.edu (S.H.); debasish@jhmi.edu (D.S.); 2 Department of Ophthalmology, The Wilmer Eye Institute, The Johns Hopkins School of Medicine, Baltimore, MD 21205, USA; szigler45@gmail.com

**Keywords:** mitophagy, mitochondria, lysosome, BCKDK, βA3/A1-crystallin, astrocytes, autophagy, optic nerve

## Abstract

Mitochondrial dysfunction in astrocytes has been implicated in the development of various neurological disorders. Mitophagy, mitochondrial autophagy, is required for proper mitochondrial function by preventing the accumulation of damaged mitochondria. The importance of mitophagy, specifically in the astrocytes of the optic nerve (ON), has been little studied. We introduce an animal model in which two separate mutations act synergistically to produce severe ON degeneration. The first mutation is in *Cryba1*, which encodes βA3/A1-crystallin, a lens protein also expressed in astrocytes, where it regulates lysosomal pH. The second mutation is in *Bckdk*, which encodes branched-chain ketoacid dehydrogenase kinase, which is ubiquitously expressed in the mitochondrial matrix and involved in the catabolism of the branched-chain amino acids. BCKDK is essential for mitochondrial function and the amelioration of oxidative stress. Neither of the mutations in isolation has a significant effect on the ON, but animals homozygous for both mutations (DM) exhibit very serious ON degeneration. ON astrocytes from these double-mutant (DM) animals have lysosomal defects, including impaired mitophagy, and dysfunctional mitochondria. Urolithin A can rescue the mitophagy impairment in DM astrocytes and reduce ON degeneration. These data demonstrate that efficient mitophagy in astrocytes is required for ON health and functional integrity.

## 1. Introduction

Astrocytes are the most abundant glial cells in the central nervous system and play a crucial role in maintaining optic nerve health and function [1]. The retina and the optic nerve track are among the most metabolically active tissues, requiring significant energy resources [2,3]. Proper mitochondrial function in various ocular cells is essential to ensure an adequate energy supply for the transmission of light signals [4,5]. Irregularities in mitochondrial function are commonly observed in various ocular diseases, including glaucoma [6], age-related macular degeneration (AMD) [7,8], and diabetic retinopathy (DR) [9]. Mitochondrial dysfunction in astrocytes has also been implicated in the development of neurological disorders, including Alzheimer’s disease (AD) [10], Parkinson’s disease [11], and inflammatory diseases [12,13,14]. However, the impact of mitochondrial dysfunction in astrocytes on the pathogenesis of ocular diseases largely remains unknown. To investigate this, we developed a unique animal model with two spontaneous mutations.

We previously reported two spontaneous mutations that arose in the Sprague Dawley rat [15,16,17]. The first of these, called Nuc1, has an obvious eye-specific phenotype in which heterozygotes have dense nuclear cataracts, and homozygotes have a much more severe phenotype with microphthalmia and lens rupture prior to birth [16,18]. Gene linkage studies identified *Cryba1* as the affected gene and subsequent analysis demonstrated an insertion mutation in Exon 6 that replaced an absolutely conserved glycine with 10 novel amino acids [16,17]. *Cryba1* encodes βA3/A1-crystallin, two isoforms that are members of the β/γ-crystallin family of proteins, major structural proteins of the ocular lens essential to the establishment of the organ’s critical attributes—refractivity and transparency [16]. Crystallins are known to have been recruited to the lens from proteins existing before the lens evolved with functions unrelated to their role in the lens. This gave rise to the concept of ‘gene sharing’ [19] or subsequently, ‘moonlighting’ proteins [20], where a single protein may have different functions when expressed in different cell types or under different conditions [20]. Our data suggest that, in Nuc1, the semi-dominant phenotype results from such a situation. In addition to being expressed at high abundance in the lens, *Cryba1* is also present at much lower levels in certain other ocular cell types, in particular astrocytes and retinal pigmented epithelium (RPE) cells [16]. While these cell types appear normal in Nuc1 heterozygotes, cells from homozygotes exhibit definitive defects in cell signaling networks particularly involving lysosomal function, autophagy, and the mTORC1 pathway [21]. Thus, Nuc1 is a recessive phenotype with respect to these cellular functions, but in the lens, the mutant protein is abundant and fails to fold properly, leading to aggregation and nuclear cataract formation even in heterozygotes. We have performed extensive investigations into the effects of the Nuc1 mutation in both astrocytes and RPE cells and the ultimate impact on the eye in vivo [15,22].

While maintaining a colony of Nuc1 rats, a second distinct phenotype appeared. In a litter of thirteen pups from a mating of two Nuc1 homozygotes, five pups displayed severe hindlimb dysfunction. The affected pups had a frog-like gait with hindlimb hypertonicity, hyperextension, and dorsal rotation. Their eyes did not appear different from their non-‘frogleg’ littermates. To determine whether we were dealing with a second de novo mutation or a new manifestation of Nuc1, we undertook a selective breeding approach to see if the two phenotypes could be separated. While this was complicated by the fact that we were unable to successfully breed ‘frogleg’ homozygotes with each other or with wild-type rats, we were eventually successful in separating the two phenotypes and establishing that ‘frogleg’ was the result of an independent mutation transmitted in an autosomal dominant fashion [15]. Using microsatellite markers spanning the rat genome, the affected gene was linked to rat chromosome 1q32-1q37; whole-genome sequencing within this linkage interval identified a missense mutation in the gene *Bckdk* (branched chain keto-acid dehydrogenase kinase) as the most likely cause of ‘frogleg’ [15]. BCKDK is ubiquitously expressed and is a critical component of the mitochondrial matrix, regulating levels of the branched-chain amino acids (BCAAs), leucine, isoleucine, and valine [23,24]. Specifically, BCKDK regulates the catabolism of the BCAAs by phosphorylating the rate-limiting enzyme in the pathway, BCKDH (branched-chain keto-acid dehydrogenase) [25]. When BCKDH is not phosphorylated at Ser293, its activity is unchecked, and levels of the BCAAs fall dramatically with significant pathological consequences [26]. We were able to demonstrate that the substitution of glutamine for a highly conserved glycine at position 369 of BCKDK was the responsible mutation by showing that, in ‘frogleg’ homozygotes, there was no detectable phosphorylation of Ser293 in BDKDH and that levels of the three BCAAs were markedly reduced, with no change in other amino acids [15]. A chance histological observation on the eyes of a rat homozygous for both mutations that had not been identified as such led us to the discovery that animals homozygous for both mutations (double mutant (DM)) exhibited severe degenerative effects on the inner retina and optic nerve. The optic nerve is composed of the axons of the retinal ganglion cells and is responsible for transmitting visual information from the retina to the brain. In the optic nerve, astrocytes express both *Cryba1* and *Bckdk*. Autophagy is a cellular process through which damaged cellular components, such as proteins and organelles, are delivered to lysosomes for degradation [27,28]. Selective mitochondrial autophagy, mitophagy, is a crucial quality control mechanism in the maintenance of proper mitochondrial function by preventing the accumulation of damaged mitochondria [29,30]. The importance of mitophagy, specifically in the astrocytes of the optic nerve, has been little studied. To address this question, here, we introduce a unique animal model in which two separate mutations (in *Cryba1* and *Bckdk*) act synergistically to produce severe optic nerve degeneration. While neither of the mutations in isolation has a significant effect on optic nerve structure or myelination, animals homozygous for both mutations exhibit very serious optic nerve degeneration and demyelination. We demonstrate that a simultaneous lack of functional βA3/A1-crystallin and BCKDK leads to lysosomal abnormality and dysfunctional mitochondria in astrocytes, which in turn leads to optic nerve degeneration. This finding highlights the importance of proper mitochondrial function and mitophagy in maintaining the health and function of astrocytes in the optic nerve.

## 2. Materials and Methods

### 2.1. Animals

All animal-related investigations adhered to the principles outlined in the Guide for the Care and Use of Animals (The National Academies Press, Washington, DC, USA) and received approval from the University of Pittsburgh Animal Care and Use Committee (Protocol Number: 20108281). Nuc1 was a spontaneous mutation in Sprague Dawley rats, and then the second mutation also arose spontaneously in our Nuc1 colony [15].

### 2.2. Astrocyte Cultures

The astrocytes from the optic nerve of 2-day-old rats were cultured following a previously established methodology [31].

### 2.3. Measurement of Autophagy Flux

To measure autophagy flux, cultured astrocytes from different genotypes were treated with bafilomycin A1 (BafA1) (Sigma-Aldrich, Burlington, MA, USA, B1793), an inhibitor of lysosomal acidification. The ratio of LC3-II/MAP1LC3B levels in the presence and absence of BafA1 was determined through immunoblotting, following a previously described method [31,32,33].

### 2.4. Measurement of Mitochondrial Membrane Potential and Superoxide Generation

To assess the mitochondrial membrane potential (MMP), we utilized tetramethylrhodamine methyl ester (TMRM; Thermo Fisher Scientific, Waltham, MA, USA, T668) as described before [33,34]. TMRM is a cationic dye that accumulates in the mitochondria in a membrane potential-dependent manner, making it useful for assessing mitochondrial health and function [35]. TMRM carries a positive charge, allowing it to accumulate within active mitochondria, which possess a negative charge. In contrast, depolarized or inactive mitochondria exhibit reduced membrane potential and do not retain TMRM as efficiently [36,37]. Additionally, mitochondrial superoxide levels were measured using the MitoSOX Red Mitochondrial Superoxide Indicator (Thermo Fisher Scientific, Waltham, MA, USA, M36008) according to the manufacturer’s instructions. Cells from all experimental conditions were washed with PBS and incubated with either 5 μM MitoSOX or 400 nM TMRM for 15 min at 37 °C, protected from light. The cells were washed with PBS thrice, scraped from the plate, and analyzed using a flow cytometer, BD FACS Aria III (BD Bioscience, Franklin Lakes, NJ, USA). The data were analyzed using FlowJo software (v10.6.178). Alternatively, mitochondrial superoxide generation was determined using lucigenin (bis-N-methyl acridinium nitrate; Sigma-Aldrich, Burlington, MA, USA, M8010), as previously described [38]. Briefly, a solution of 5 mM lucigenin was prepared in Hanks’ balanced salt solution (HBSS, Thermo Fisher Scientific, Waltham, MA, USA, 88284). Subsequently, 100 μL of pre-warmed lucigenin buffer was added to the culture plates and incubated at 37 °C and 5% CO_2_ for 5 min. Luminescence readings were then obtained using a Glomax bioluminescence apparatus (Promega, Madison, WI, USA, E5311), with 5 consecutive readings per sample taken for 1 s each. The luminescence signal was adjusted based on the total protein content and expressed as arbitrary units (AUs) per milligram of protein.

### 2.5. Viral Delivery of COX8–EGFP–mCherry 

For the generation of COX8–EGFP–mCherry, the pCLBW COX8 EGFP mCherry plasmid was procured from Addgene (Watertown, MA, USA, 78520), which was initially deposited by Dr. David Chan’s laboratory. Subsequently, the sequence encoding COX8–EGFP–mCherry was subcloned into an adenovirus vector by employing the services of Vector Biolabs, Malvern, PA, USA. To ensure the expression of the construct, cells were infected with the adenovirus at a multiplicity of infection (MOI) of 50 and allowed to incubate for 24 h before commencing the experiments [33,39].

### 2.6. Western Blotting and Quantification

Western blotting and quantification were conducted following the previously established protocols [33]. The signal intensity of the detected protein was normalized to that of the housekeeping protein and analyzed using ImageJ analysis software (latest version 1.53t). All targeted proteins and internal loading controls were detected and confirmed to be within the same linear range. The primary antibodies used for western blotting in this study were as follows: MBP from Biolegend (San Diego, CA, USA (808401)); LC3, phospho-P70S6K, P70S6K, phospho-S6, S6, and cleaved caspase 1 from Cell Signaling Technology (Danvers, MA, USA (2775, 9204, 2708, 2211, 2217, and 89332)); SOD2, CNPase, and vinculin from Abcam (Waltham, MA, USA (ab13533, ab6319, and ab129002)); SQSTM1/p62 and NLRP3 from Novus Biologicals (Englewood, CO, USA (NBP1-42821 and NBP2-12446)); and β-Actin from Sigma-Aldrich (Burlington, MA, USA (A2066)). The secondary antibodies used were peroxidase-labeled goat anti-rabbit and anti-mouse antibodies from KPL (Sera Care, Milford, MA, USA, (074–1506 and 074–1806)).

### 2.7. Enzyme-Linked Immunosorbent Assay (ELISA)

ELISA was conducted utilizing lysates from 4-month-old optic nerves, following previously established procedures [40]. In brief, the optic nerves were homogenized in 300 μL of complete extraction buffer (Abcam, Waltham, MA, USA, ab193970). Subsequently, the ELISA was performed in 96-well microtiter plates (Sigma-Aldrich, Burlington, MA, USA, M9410-1CS) coated with tissue lysates and then left to incubate overnight at 4 °C. The plates were subsequently blocked with 5% BSA for 2 h. After PBS washing, 50 μL of IL1β antibody (Abcam, Waltham, MA, USA, ab9722) at a 1:1000 dilution was added to each well and allowed to incubate for 2 h at room temperature. The cytokines bound to the plate were detected using secondary IgG-HRP (KPL, Sera Care, Milford, MA, USA, 074–1506). TMB substrate solution (Thermo Fisher Scientific, Waltham, MA, USA, 34021) was used to develop color, and the reaction was stopped with 2 N H_2_SO_4_ solution (Thermo Fisher Scientific, Waltham, MA, USA, MK-H381-1). The absorbance was measured at 450 nm using a microplate reader [40].

### 2.8. Hematoxylin–Eosin Staining

The eyes of rats from different genotypes underwent the following procedure for hematoxylin–eosin staining as described before [18]: The initial fixation of the eyes was accomplished using a solution containing 2.5% glutaraldehyde (Sigma-Aldrich, Burlington, MA, USA, G5882) followed by formalin treatment. The fixed eyes were subsequently subjected to a series of graded ethanol solutions, progressively increasing in concentration. This process aimed to facilitate dehydration. The eyes were then embedded in methyl methacrylate (Sigma-Aldrich, Burlington, MA, USA, M55909), and 1 μm sections were cut. These sections were then subjected to hematoxylin and eosin staining (TissuePro Technology, Gainesville, FL, USA, EY07-500R) and were examined utilizing a light microscope, enabling the detailed observation of the cellular structures and characteristics.

### 2.9. Electron Microscopy

The rat optic nerves from different genotypes underwent the following preparation steps for electron microscopy as explained previously [41]: The optic nerves were initially fixed in a solution containing 1.5% glutaraldehyde in 0.08 mol/L phosphate buffer (Sorenson’s buffer, Thermo Fisher Scientific, Waltham, MA, USA, 50365831) at pH 7.2. This fixation process was conducted for a duration of 2 h at room temperature. After fixation, the nerves underwent two 10 min washes in the buffer. A post-fixation procedure was performed using 1% osmium tetroxide for 1 h. A series of alcohol washes were carried out to dehydrate the nerves. Subsequently, the nerves were transitioned to propylene oxide. The nerves were infiltrated with a mixture of 50% propylene oxide (Sigma-Aldrich, Burlington, MA, USA, 75569) and Epon resin (GracoRoberts, Arlington, TX, USA, V00298) overnight. Ultrathin sections were prepared utilizing a Leica Ultramicrotome UCT (Leica Microsystems, Wetzlar, Germany). These sections were subsequently stained with uranyl acetate and lead citrate before being visualized using an H7600 transmission electron microscope (Hitachi, Tokyo, Japan).

### 2.10. Drug Delivery to Animals 

UA (Sigma-Aldrich, Burlington, MA, USA, SML1791) was initially prepared at a concentration of 25 mg/mL in DMSO, dimethyl sulfoxide (Thermo Fisher Scientific, Waltham, MA, USA, J66650.AK). This concentrated solution was then appropriately diluted using PBS (pH = 7.4) to attain a final concentration of 0.25 mg/mL. The final DMSO concentration that was used as vehicle control in any treatment was 1%. DM rats were intraperitoneally (i.p.) treated with the prepared UA solution at a dose of 5 mg/kg body weight. In addition, 21-day-old WT and Nuc1 rats received intraperitoneal injections of UA or 1% DMSO (as the vehicle) three times a week over a span of three months. Following this treatment regimen, the optic nerves were fixed for immunohistochemical and immunoblotting analyses.

### 2.11. Tissue Preparation and Immunohistochemistry

Standard techniques were used for tissue preparation and subsequent immunohistochemistry analysis, following the established protocol as described [42,43]. Images from sections immunostained for Iba1 (Abcam, Waltham, MA USA, ab178846), RPBMS (Abcam, Waltham, MA USA, ab152101), and CD4 (Abcam, Waltham, MA USA, ab133616) were captured using a Zeiss confocal microscope. The obtained images were then subjected to analysis using the Zen Light Edition 2009 software (ZEN 2009-Zeiss Enhanced Navigation), which is a free digital image processing software offered by Zeiss for this purpose.

### 2.12. Statistical Analysis

GraphPad 6.0 software was utilized for conducting statistical analysis. The significance levels (*p*-values) were determined through 2-tailed Student’s *t*-test and repeated-measure ANOVA tests, followed by Tukey’s post hoc test. These analyses were based on data collected from a minimum of three distinct experiments. Statistical significance between the groups was established as follows: * for *p* < 0.05, ** for *p* < 0.01, and *** for *p* < 0.001. Data are expressed as mean ± SEM, and each biological replicate consists of a minimum of three technical replicates.

## 3. Results

### 3.1. Optic Nerve Degeneration in the DM Rats

**Figure 1:**
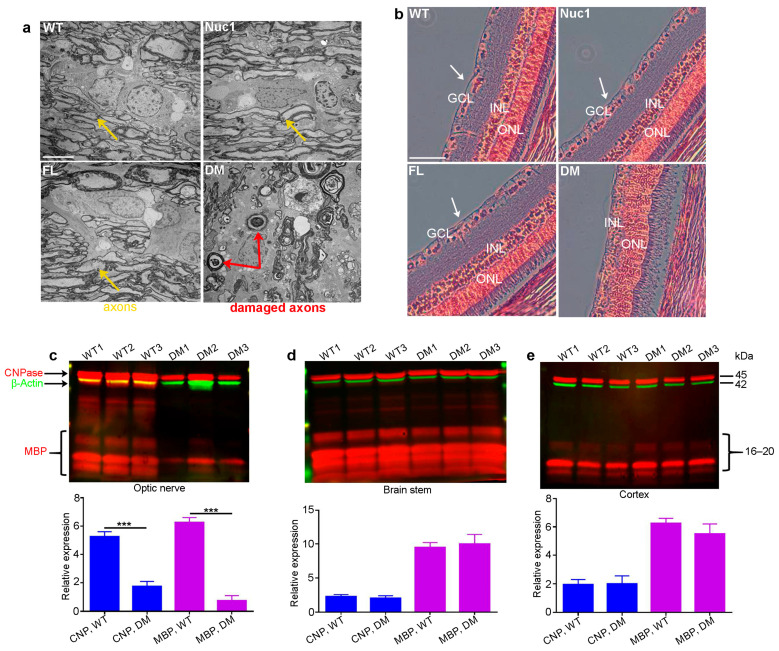
Degeneration of optic nerve in DM rats: (**a**) Transmission electron microscopy (TEM) revealed severe optic nerve degeneration in the DM rats compared to other genotypes at 4 months of age. Orange and red arrows show the axons and damaged axons, respectively. Bar: 5 µm. (**b**) H&E staining showing loss of RGCs (white arrows) in 4-month-old DM rats relative to other genotypes. GCL: ganglion cell layer; INL: inner nuclear layer; ONL: outer nuclear layer, Bar: 100 µm. (**c**) The levels of both CNPase and MBP are significantly decreased specifically in the optic nerves of DM rats at 4 months of age compared to WT, but (**d**) the brainstem and (**e**) cortex are not affected. Graphs represent mean ± SEM from at least 3 independent experiments repeated in triplicate, *** *p* < 0.001.

Eyes and optic nerves were prepared for histological analysis from groups of wild-types (WT), Nuc1 homozygous (Nuc1), frogleg homozygous (FL), and double-mutant (DM) homozygous rats at 4 months of age. Optic nerve cross-sections for DM show massive degeneration on TEM images, while the Nuc1 and FL samples have an optic nerve structure similar to WT (Figure 1a). Likewise, cross-sections of the retinas clearly show the absence of the inner nuclear layer (ganglion cells) in the DM sample, while both single mutants appear essentially normal (Figure 1b). To corroborate these results and determine if the myelination defects seen in the optic nerve are selective for that tissue, extracts were prepared from optic nerves and the brainstem and cortex (other heavily myelinated tissues) in groups of WT and DM rats and analyzed via western blotting for markers of myelination (MBP-myelin basic protein and CNPase-2’,3’-Cyclic-nucleotide 3’-phosphodiesterase) [44,45]. Figure 1c shows that while both markers are highly significantly reduced in the DM optic nerve relative to WT, no such difference is seen in the brain stem or cortex (Figure 1d,e) at 4 months of age. Thus, the myelination defect seems to be specific to the optic nerve.

### 3.2. Optic Nerve Forms Normally in the DM and then Degenerates

**Figure 2:**
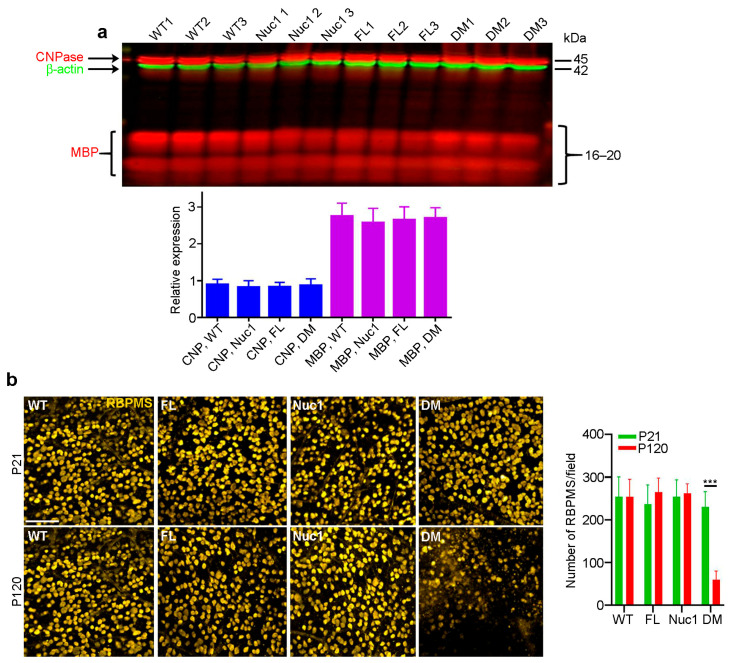
Optic nerves form normally in the DM and then degenerate: (**a**) In contrast to the changes shown in Figure 1c, at P21, immunoblotting showed that there were no differences in levels of CNPase or MBP in optic nerves of DM rats relative to the other genotypes. (**b**) Immunohistochemistry of retinal flat mounts against RBPMS (anti-RNA binding protein with multiple splicing) was used to detect RGCs. RBPMS staining was significantly reduced in DM retinas relative to other genotypes at 4 months of age. Bar: 50 µm. Graphs represent mean ± SEM from at least 3 independent experiments repeated in triplicate, *** *p* < 0.001.

In the rat optic nerve, myelination is generally completed by 3–4 weeks after birth [46]. To determine whether the myelination deficits seen in the DM optic nerves at 4 months reflected a failure to produce myelin or a degeneration of myelin after it was produced, we repeated the above analyses on 21-day-old rats. As seen in Figure 2a, at 21 days of age, all four genotypes have similar levels of both myelin markers. As could be expected, staining for retinal ganglion cells on retinal flat mounts from rats of all four genotypes at 21 days and 4 months of age showed cell loss only in the 4-month DM, with no apparent change at 21 days relative to the WT and single-mutant samples (Figure 2b). This is consistent with the great reduction in MBP in the optic nerve of 4-month DM rats, which is not observed in 21-day-old rats. These data demonstrate that the loss of myelin in the DM optic nerve results from degeneration after myelination is complete.

### 3.3. Optic Nerve Degeneration Is Associated with Inflammation

**Figure 3:**
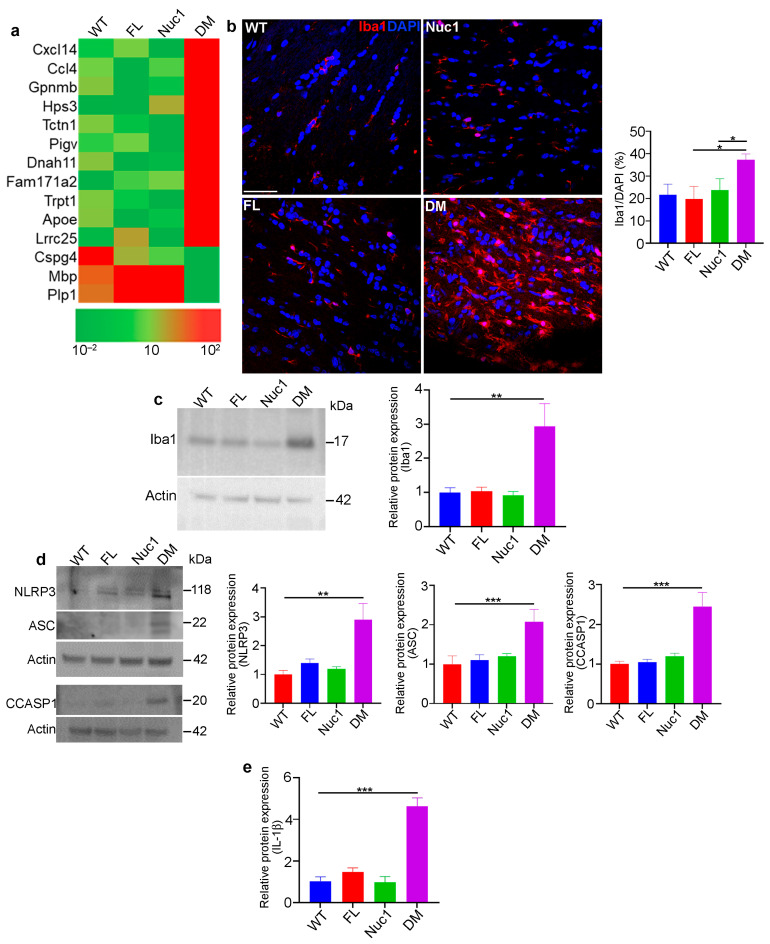
Inflammation in the optic nerve of DM rats: (**a**) RNA-Seq analysis of 4-month-old optic nerves revealed changes in the mRNA expression pattern of several inflammatory factors in DM relative to other genotypes. (**b**,**c**) Immunohistochemical staining and western blotting of optic nerve sections from 4-month-old rats against Iba1 (microglia/macrophages) showed increased staining and protein levels in DM rats relative to other genotypes. Bar: 100 µm. (**d**) Representative immunoblotting images demonstrate that expression of inflammasome markers including NLRP3, ASC, and cleaved caspase 1 were significantly increased in DM optic nerve relative to the other genotypes. (**e**) Measuring IL-1β using ELISA showed a significant increase in DM optic nerve relative to other genotypes. Values are plotted as mean ± SEM from 3 independent experiments repeated in triplicate, * *p* < 0.05; ** *p* < 0.01; *** *p* < 0.001.

The RNA-Seq analysis of optic nerves from 4-month-old rats revealed increases in the expression of several important inflammation-associated genes in DM compared to the other genotypes. However, the expression of myelin/oligodendrocytes-related genes (Mbp, Plp1, and Cspg4) was significantly reduced in DM (Figure 3a). To determine whether inflammation is involved in DM optic nerve degeneration, the microglia/macrophage populations in the four different genotypes were characterized. To this end, optic nerves were dissected from 4-month-old rats and analyzed via immunostaining and immunoblotting for ionized calcium binding adaptor molecule 1 (Iba1, marker of microglia/macrophages) [47]. DM rats demonstrated a drastic increase in the number of Iba1-positive cells, compared to the other genotypes (Figure 3b,c). Furthermore, the immunostaining of optic nerves from different genotypes for CD4 led to the detection of CD4+ T lymphocytes [48] only in DM optic nerve (Figure S1). The activation of microglia/macrophages is also associated with the activation of the inflammasome pathway, a critical component of the immune response in the central nervous system. Its activation in both astrocytes and microglia can contribute to neuroinflammation and the pathogenesis of neurological disorders [49,50]. We found that the expression of inflammasome markers was significantly increased only in the DM optic nerves (Figure 3d,e). The inflammasome pathway produces the bioactive form of IL-1β, a crucial cytokine for both immunoregulation and inflammation [51,52]. We found that the expression of IL-1β is increased in DM optic nerves, indicating that degeneration of the optic nerve in DM rats is associated with the activation of inflammation.

### 3.4. Excessive Mitochondrial Oxidative Stress in DM Astrocytes

**Figure 4:**
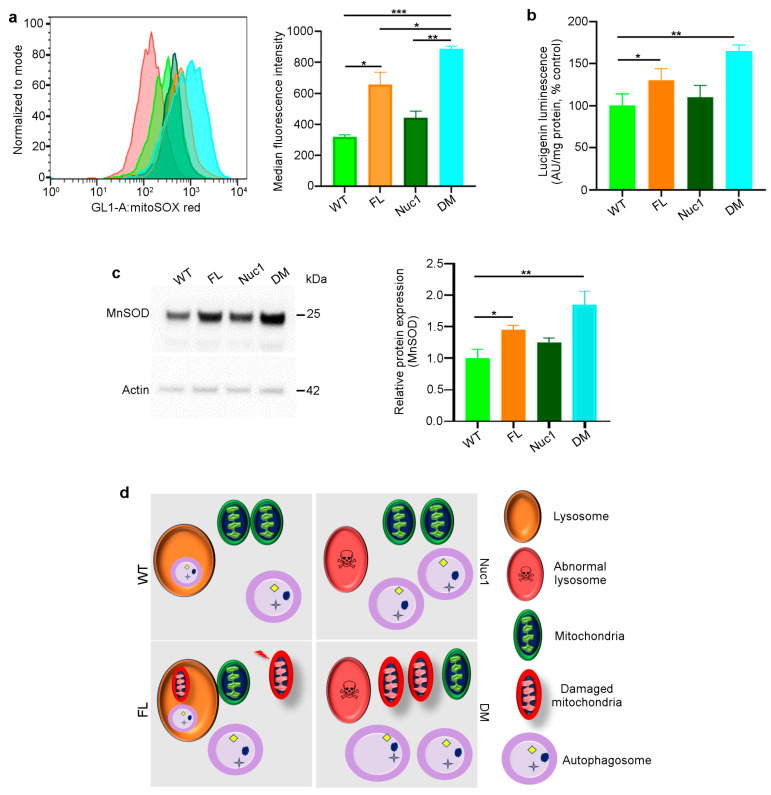
Mitochondrial superoxide production is increased in DM astrocytes: (**a**) Representative histograms of flow cytometric analysis of MitoSOX fluorescence in different experimental groups show that the expression of MitoSOX is increased in FL and DM astrocytes. (**b**) Quantitative analysis of superoxide release indicates that the level of superoxide is increased in FL and DM astrocytes relative to WT control cells. (**c**) Immunoblotting of proteins extracted from astrocytes showed elevated MnSOD expression in FL and DM genotypes relative to WT as control. (**d**) The proposed model of the astrocyte abnormality in DM rats. In WT astrocytes, mitochondria and lysosomes are healthy; thus, autophagosomes are digested in lysosomes. In Nuc1 astrocytes, lysosomes are abnormal, and autophagosome degradation is impaired. In FL astrocytes, oxidative stress leads to mitochondrial damage; however, lysosomes and the mitophagy process are normal. In DM astrocytes, lysosomes are abnormal, so mitophagy is not completed, leading to the accumulation of damaged mitochondria. Data are mean ± SEM, * *p* < 0.05; ** *p* < 0.01; *** *p* < 0.001.

Given that astrocytes are the only cells in the optic nerve that express both *Cryba1* and *Bckdk*, we investigated the synergistic effects of the two mutations on astrocyte function. Astrocytes were isolated from adult rats (all four different genotypes) and expanded in culture. The MitoSox assay [33,53] demonstrated increased mitochondrial reactive oxidative stress in FL and particularly DM astrocytes (Figure 4a). In addition, mitochondrial superoxide anion levels were significantly increased in FL and DM astrocytes relative to WT and Nuc1 cells (Figure 4b). The expression of mitochondrial superoxide dismutase 2, (SOD2) [33,54], a mitochondrial antioxidant, was significantly increased in FL and DM astrocytes relative to other genotypes, consistent with increased oxidative stress in FL and DM cells (Figure 4c). In addition, the mitochondrial membrane potential (MMP) was significantly reduced only in DM astrocytes relative to the other genotypes (Figure S2). These data collectively show that oxidative stress affects the function of mitochondria in DM astrocytes to a much greater extent than in astrocytes from single-mutant animals.

This information led us to speculate and envision the following mechanism for the generation of the astrocyte abnormality in the DM optic nerve (Figure 4d): In astrocytes derived from WT rats, we expect to see normal lysosomes and mitochondria, as well as basal levels of mitophagy and oxidative stress. In Nuc1 rats, the previously documented lysosomal abnormality is expected to result in impairment of autophagosome digestion, leading to a reduction in autophagy flux, with the health of mitochondria being unaffected. In FL animals, oxidative stress is expected to result in mitochondrial damage [24] that would be counteracted by increased mitophagy. However, in DM rats, the mitochondria, damaged from the *Bckdk* mutation, are expected to accumulate due to the inability of abnormal lysosomes (caused by the *Cryba1* mutation) to complete the mitophagy process. The accumulation of damaged mitochondria would also be expected to significantly reduce MMP in DM astrocytes.

### 3.5. Autophagy Flux Is Impaired in DM Astrocytes

**Figure 5:**
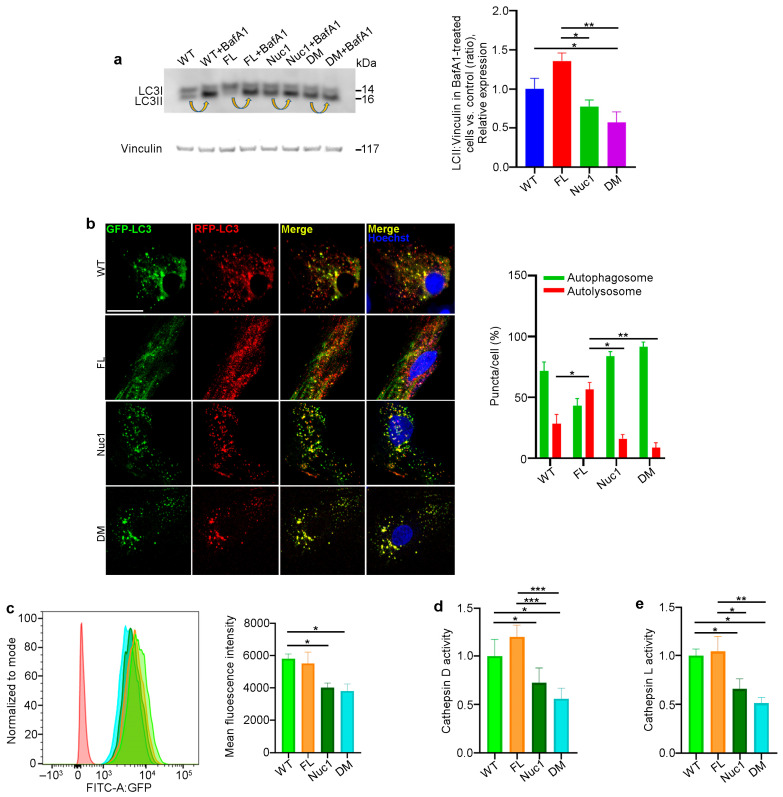
Autophagy is diminished in DM but not in FL astrocytes: (**a**) Astrocytes from the different genotypes were cultured in the presence or absence of the lysosomal inhibitor bafilomycin A1 (BafA1). After treatment, the cells were lysed, and protein lysates were analyzed for LC3 via immunoblotting. There was a significant decrease in the accumulation of the autophagosome-positive LC3-II isoform in DM cells compared to WT and FL cells after BafA1 treatment. Arrows show the accumulation of LC3-II after BafA1 treatment in each cell type. This suggests that the DM cells have a defect in autophagy, which may have caused the reduced accumulation of LC3-II. (**b**) Astrocytes from all genotypes were transduced using an RFP–GFP tandem fluorescent-tagged LC3 (RFP–GFP–LC3) construct. Yellow puncta indicate the presence of autophagosomal structures, as both GFP and RFP fluoresce at the cytoplasmic pH. In contrast, red puncta signify autolysosomes, as the acidity of lysosomes quenches the GFP fluorescence. Merged confocal images show that FL astrocytes have many red puncta, indicating the presence of autolysosomes, while Nuc1 and DM cells have mostly yellow puncta, indicating a blockage of autophagic flux. The numbers of autophagosomes and autolysosomes were quantified from 30 images per group for each experiment. (**c**) Representative histograms of flow cytometric analysis of LysoSensor fluorescence in different experimental groups show that the fluorescent intensity of LysoSensor is reduced in DM astrocytes relative to WT cells. (**d**,**e**) The graphs show that the levels of active cathepsin D and L were significantly reduced in DM astrocytes. Data are mean ± SEM; n = 3, * *p* < 0.05. Scale bar: 20 μm. Data are mean ± SEM; n = 30 cells from three different experiments, * *p* < 0.05; ** *p* < 0.01; *** *p* < 0.001.

Autophagy is a dynamic process, and its activity is described as ‘autophagy flux’ [55]. LC3, a key protein in the autophagy pathway, is proteolytically cleaved to form LC3I and then lipidated to generate LC3-II. LC3-II is a marker of autophagosomes and is degraded in lysosomes; therefore, LC3-II turnover is an indicator of autophagy flux [56,57]. A standard method of measuring autophagy flux is immunoblotting for LC3-II in the presence and absence of bafilomycin A1 (BafA1), a lysosomotropic agent that suppresses lysosomal acidification, thereby inhibiting lysosomal function. The LC3-II level in BafA1-treated cells is divided by the level of LC3-II in untreated cells [58]. Astrocytes from all four different genotypes were cultured in the presence and absence of 50 nM BafA1 for 3 h; then, the protein was extracted and analyzed for LC3-II via western blotting to determine autophagy flux. We found that the level of LC3-II accumulation in Nuc1 and DM astrocytes was significantly reduced relative to FL and WT cells, indicating an impairment in autophagy flux in these cells (Figure 5a). These data confirm our previous findings that demonstrated lysosomal abnormality and autophagy impairment in Nuc1 astrocytes [31,59]. In addition, to validate the autophagy impairment in DM astrocytes, these cells were transduced with a tandem repeat red fluorescent protein (RFP)–green fluorescent protein (GFP)–LC3B (microtubule-associated protein 1 light chain 3 beta) construct to show the levels of autolysosomes relative to autophagosomes. By fusing an acid-sensitive GFP with an acid-insensitive RFP, the transition from autophagosome (neutral pH) to autolysosome (acidic pH) was analyzed through the selective disappearance of GFP fluorescence [60]. The ratio of autolysosomes to autophagosomes, which is an indicator of autophagy flux [55], was significantly increased in FL astrocytes and reduced in both Nuc1 and DM astrocytes relative to WT (Figure 5b). This demonstrates that autophagy flux is significantly increased in FL astrocytes relative to astrocytes from the other genotypes. In contrast, the level of autophagy flux is greatly suppressed in DM.

Considering the fact that proper lysosomal function is essential for autophagy machinery and that the loss of *Cryba1* gene expression impairs lysosomal function in Nuc1 and DM astrocytes, we compared the function of lysosomes in the different genotypes. To measure lysosomal function, live cells were stained with LysoSensor as an indicator of lysosomal pH. The fluorescence of the LysoSensor dye increases in acidic environments [61]. We found that WT and FL astrocytes with normally acidic lysosomes displayed increased LysoSensor fluorescence. However, LysoSensor fluorescence intensity was significantly reduced in Nuc1 and DM astrocytes, indicating an abnormally elevated lysosomal pH in these cells (Figure 5c). In addition, the function of lysosomes was evaluated by measuring the activities of the lysosomal enzymes cathepsin D and L [62,63]. Cathepsins are a family of protease enzymes primarily found in lysosomes that are active in a low-pH environment [64]. We found that the activities of both cathepsin D and L were significantly reduced in DM and Nuc1 cells relative to WT and FL cells (Figure 5d,e).

### 3.6. Mitophagy Is Impaired in DM Astrocytes

**Figure 6:**
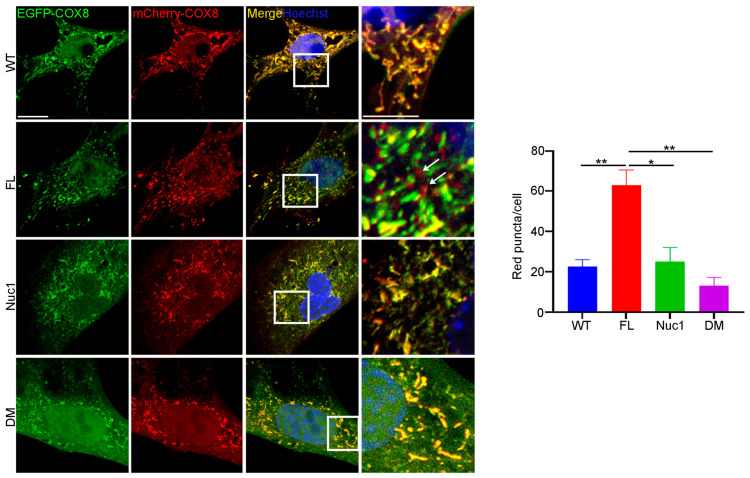
Mitophagy is impaired in DM astrocytes. Astrocytes from all genotypes were transduced by adenovirus–COX8–EGFP–mCherry to label mitochondria and observed under a confocal microscope. The number of acidic (red) mitochondria was significantly increased in FL astrocytes and greatly diminished in DM astrocytes relative to other genotypes. Scale bar: 20 μm. Data are mean ± SEM; n = 30 cells from three different experiments. * *p* < 0.05; ** *p* < 0.01.

Since autophagy flux was dysregulated, and mitochondrial oxidative stress was increased in DM astrocytes relative to FL, we wondered if the clearance of abnormal mitochondria (mitophagy) is also impaired in the DM astrocytes. To measure the level of mitophagy, a fluorescent reporter COX8 (cytochrome c oxidase subunit 8)-enhanced GFP (EGFP)–mCherry fluorescence reporter that is targeted to the mitochondrial matrix was used [65]. Since EGFP is sensitive to pH, the red puncta correspond to mitochondria in lysosomes because EGFP is quenched by the acidity of lysosomes. The yellow puncta represent normal mitochondria because both EGFP and mCherry fluoresce at cytoplasmic pH. Therefore, the number of red puncta in each cell is a measure of mitophagy. We found that the number of red puncta was greatly reduced in DM astrocytes relative to FL. However, the number of red puncta was significantly increased in FL astrocytes relative to the other genotypes, suggesting that FL astrocytes contain many abnormal mitochondria that are being actively removed through mitophagy (Figure 6). We did not see a difference in mitophagy between WT and Nuc1 astrocytes, most likely because mitochondria are normal in these cells.

### 3.7. mTORC1 Is Overactivated in Cultured DM Astrocytes and Optic Nerve

**Figure 7:**
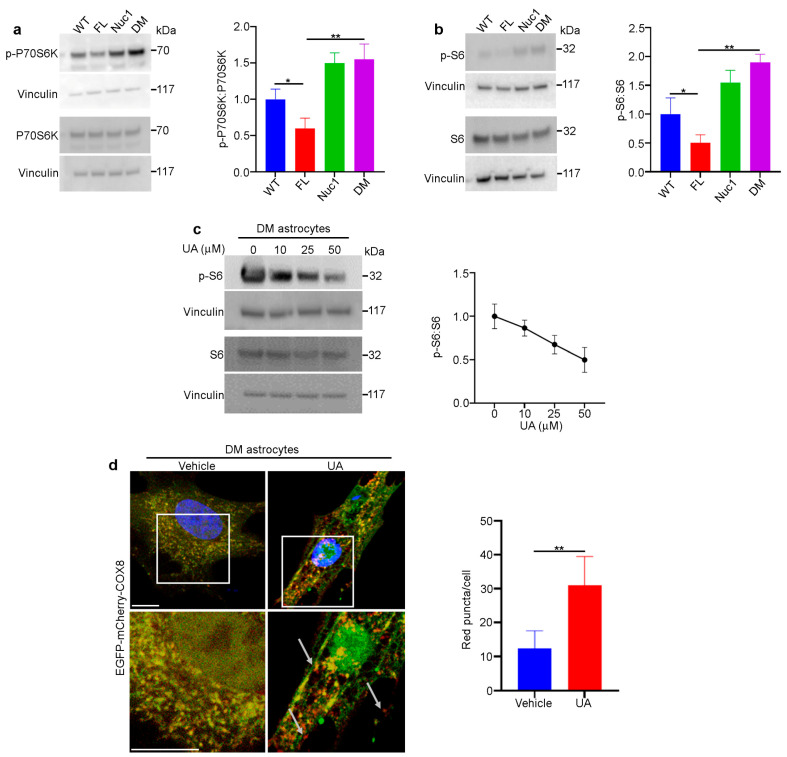
The mTORC1 pathway is overactivated in DM astrocytes: (**a**,**b**) Overactivation of the mTORC1 pathway in DM astrocytes. Protein lysates of cultured astrocytes were prepared and analyzed via immunoblotting for activation markers of the mTORC1 pathway. The DM and Nuc1 astrocytes showed a significant increase in two different phosphorylated (active) forms of RPS6KB1 and phosphorylated-RPS6 compared to the WT group. In contrast, the FL astrocytes showed a significant reduction in these activated molecules. The levels of total RPS6 were not significantly different among the different groups. The presented graphs show the ratios of phosphorylated proteins (active forms of RPS6KB1 and phosphorylated-RPS6) to total protein in the different genotypes. (**c**) Representative western blot images and densitometry showed that UA treatment reduced the ratio of PS6:S6 in DM astrocytes in a dose-dependent manner. Vinculin was used as a loading control. (**d**) DM astrocytes were transduced using a GFP–mCherry–COX8 construct and then treated with 100 µM UA for 24 h. The representative confocal images showed that the number of red puncta (mitochondria) increased with UA treatment (n = 30). White arrows show the red puncta. Values are plotted as mean ± SEM from 3 independent experiments repeated in triplicate. * *p* < 0.05; ** *p* < 0.01.

We have previously shown that the loss of functional βA3/A1-crystallin in Nuc1 astrocytes leads to the upregulation of mTORC1, a negative regulator of autophagy [31]. In addition, BCKDK is also important in the regulation of cellular oxidative stress that in turn affects mTORC1 function. Therefore, we wanted to know if the lack of both functional *Cryba1* and *Bckdk* genes has a synergistic effect on the mTORC1 pathway in DM astrocytes. Consistent with our previous findings, we found that the level of mTORC1 signaling was increased in Nuc1 astrocytes. However, the level of mTORC1 activity was reduced in FL astrocytes, most likely because of dysregulation in the metabolism of BCAAs (Figure 7a,b). Interestingly, mTORC1 signaling in DM astrocytes was increased as in Nuc1 cells. Previous research has demonstrated that βA3/A1-crystallin controls the localization of mTORC1 to lysosomes by binding to V-ATPase, which is the crucial proton pump that acidifies lysosomes [16]. To confirm the in vitro observations, optic nerves from different genotypes were isolated and protein lysates were analyzed via immunoblotting. We found that the expression of SQSTM1 [66] was significantly increased in the Nuc1 and DM optic nerves, indicating the impairment of autophagy; however, it was reduced in FL optic nerves, indicating increased autophagic degradation. These data are consistent with in vitro data showing that autophagy flux is reduced in DM astrocytes. In addition, we also found that autophagy impairment is associated with mTORC1 overactivation in DM and Nuc1 optic nerves (Figure S3a,b).

### 3.8. Urolithin A (UA) Rescues Autophagy Impairment in DM Astrocytes

UA is produced by gut microflora from the transformation of ellagitannins that are found naturally in plants such as walnuts, pomegranates, raspberries, and strawberries [67,68]. UA treatment helps to maintain mitochondrial quality control by stimulating mitophagy and preventing the accumulation of damaged mitochondria [69,70]. It has been shown that UA induces autophagy by inhibiting the Akt/mTORC1 signaling pathway [71]. The treatment of DM astrocytes with UA leads to the inhibition of the mTORC1 signaling pathway and increased autophagy flux (Figure 7c and Figure S4). In addition, the mitophagy abnormality in DM astrocytes was rescued using UA treatment (Figure 7d).

### 3.9. UA Treatment Mitigates Optic Nerve Degeneration in DM Rats

**Figure 8:**
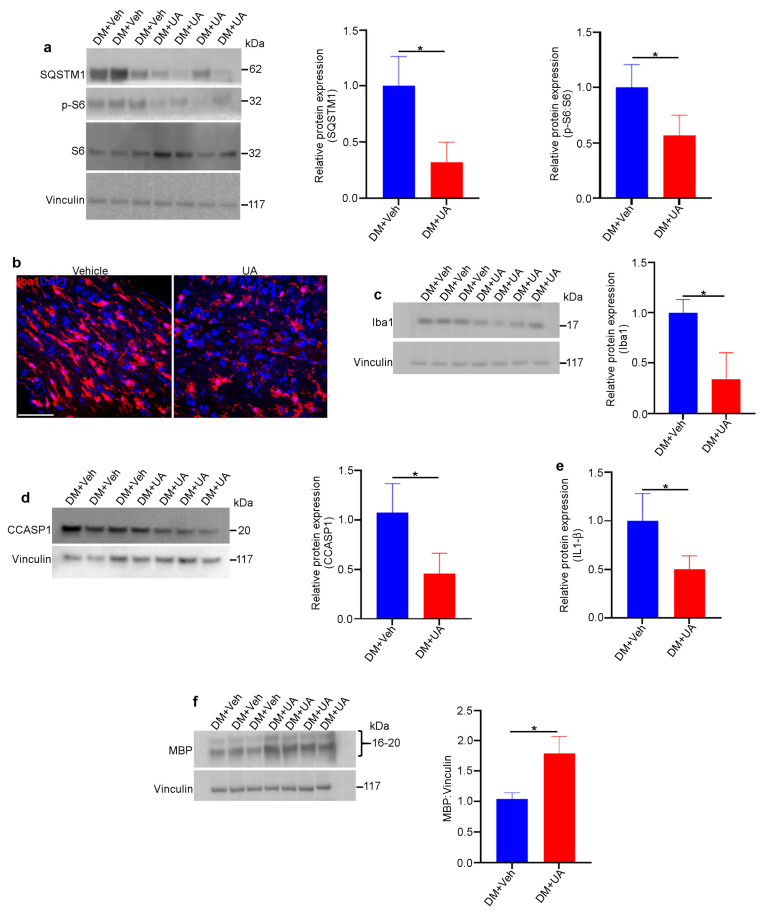
UA protects DM optic nerves against degeneration. Rats from different genotypes were treated with UA from P21 to P120. Then, optic nerve protein lysates were analyzed via immunoblotting: (**a**) Representative immunoblotting images showed that the expression of SQSTM1 and the ratio of PS6:S6 were reduced in the optic nerves of UA-treated DM rats. (**b**) Representative confocal microscopy images revealed that Iba1 staining was decreased via UA treatment. Scale bar: 100 μm. (**c**) Immunoblotting of tissue lysates confirmed the reduction in Iba1. (**d**) Western blotting showed that the expression of cleaved caspase 1 (CCASP1) was reduced in UA-treated DM optic nerves. (**e**) Analysis of IL-1β in optic nerve lysates indicated that IL-1β was reduced by UA treatment; n = 6. (**f**) Western blotting analysis showed that the expression of MBP was increased in DM optic nerves after UA treatment. Vinculin was used as a loading control. Values are given as mean ± SEM from 3 independent experiments, * *p* < 0.05.

UA can cross the blood–brain barrier [72] and has been shown to be a powerful neuroprotectant in vivo against various neurodegenerative diseases [73]. The intraperitoneal injection of UA guaranteed the presence of UA in circulation [74]. In this study, UA was administered within the time frame when optic nerve degeneration occurred in DM rats. UA (or vehicle) was injected intraperitoneally into DM rats three times per week starting at one month of age and continuing for three months. We found that UA treatment led to a reduction in mTORC1 pathway activity and decreased levels of SQSTM1, indicating increased autophagy in DM optic nerves (Figure 8a). In addition, the population of microglia/macrophages was significantly reduced by UA treatment, as was the expression of inflammasome pathway markers (Figure 8b–e). Finally, the level of MBP expression in the DM optic nerves was significantly increased through UA treatment (Figure 8f). Given the fact that MBP is significantly diminished as a result of optic nerve degeneration in DM rats, UA treatment led to a significant reduction in myelin loss and therefore the progression of optic nerve degeneration in DM rats.

## 4. Discussion

Our study shed light on the connection between mitophagy in astrocytes and optic nerve health. First, we introduced a rat animal model with mitochondrial abnormality in the optic nerve astrocytes. Second, we found that impairment in mitophagy and mitochondrial health in optic nerve astrocytes leads to optic nerve degeneration. Finally, UA rescues the autophagy/mitophagy impairment in optic nerve astrocytes and inhibits the degeneration of the optic nerve in DM rats. Therefore, our studies reveal that optic nerve health profoundly relies on mitochondrial health in astrocytes.

It has been shown that a lack of *Bckdk* causes mitochondrial abnormalities and the production of excessive oxidative stress [24]. Consistently, we also found that mitochondrial oxidative stress was increased in both FL and DM astrocytes. However, the mitochondrial oxidative stress was greater in DM astrocytes than in FL astrocytes. The oxidative stress was associated with a reduction in MMP in DM but not in FL astrocytes.

It has been previously shown that βA3/A1-crystallin regulates lysosomal function and modulates signaling molecules in the lysosomes of astrocytes [16]. We also found that a lack of functional *Cryba1* in Nuc1 astrocytes leads to lysosomal abnormality and autophagy impairment [31]. The elimination of damaged mitochondria through mitophagy is required for cellular homeostasis [75,76]. As our data demonstrate, in FL astrocytes, functional lysosomes and an efficient mitophagy process eliminate damaged mitochondria caused by the lack of *Bckdk*. However, in DM astrocytes, the process of mitophagy is not completed because of lysosomal abnormality, and it leads to the accumulation of damaged mitochondria and excessive mitochondrial oxidative stress (Figure 4d). Autophagy impairment does not lead to optic nerve degeneration in Nuc1 rats. However, the coincidence of mitochondrial abnormality with dysfunctional lysosomes in DM astrocytes leads to the accumulation of damaged mitochondria and excessive oxidative stress in astrocytes and consequently optic nerve degeneration. This evidence indicates that mitophagy in astrocytes is essential for the homeostasis of the optic nerve. Moreover, optic nerve degeneration in this animal model emphasizes that retinal ganglion cells are highly metabolic cells and very sensitive to inadequate astrocyte-derived nutrient supply [5].

Lysosomes were once thought to be simply a bag of waste disposal enzymes [77], but they are now recognized as a center for multiple signaling pathways, particularly the mTORC1 pathway, a master negative regulator of autophagy [78]. V-ATPase is a proton pump in lysosomes that is responsible for maintaining the acidic pH of the lysosomal lumen and plays an important role in the activation and deactivation of mTORC1 [16,79]. Furthermore, studies have shown that βA3/A1-crystallin interacts with V-ATPase and that the lack of *Cryba1* leads to lysosomal dysfunction and overactivation of the mTORC1 pathway [16]. In addition, it is known that mitochondrial oxidative stress inhibits mTORC1 activity [80,81], and this is likely the cause of the inhibited mTORC1 pathway and the increase in lysosomal function in FL astrocytes. In DM astrocytes, mTORC1 is overactivated, similar to Nuc1. This indicates that in DM astrocytes, lysosomal abnormality caused by the *Cryba1* deficiency prevents oxidative stress-mediated mTORC1 inhibition. Therefore, inhibiting the mTORC1 pathway in DM astrocytes can be a strategy for rescuing impaired autophagy and mitophagy.

It has been shown that UA induces autophagy/mitophagy by inhibiting the mTORC1 pathway in a variety of experimental settings [67,69,74]. Our data also demonstrated that the treatment of DM astrocytes with UA led to the inhibition of mTORC1 and the rejuvenation of impaired autophagy/mitophagy. Therefore, we postulate that mTORC1 inhibition by the mitophagy/autophagy inducer UA can be a strategy to inhibit optic nerve degeneration in DM rats. It has been shown that UA improves mitochondrial function, induces mitophagy, and reduces neurodegeneration in various animal models of AD [82,83]. Our data showed that UA inhibits the degeneration of DM optic nerves by inhibiting mTORC1 and inducing autophagy. In addition, it has been shown that UA reduces neuroinflammation and improves cognitive function in a mouse model of AD [84]. Our data showed that optic nerve degeneration in DM rats is associated with the activation of microglia and inflammation. We also found that UA targets inflammasome activation in microglia/macrophages and attenuates optic nerve degeneration in DM rats.

## 5. Conclusions

Mitochondrial dysfunction is linked to various ocular diseases, including autosomal dominant optic atrophy [85], Leber’s hereditary optic neuropathy [86], glaucoma [87], age-related macular degeneration (AMD) [88], and diabetic retinopathy (DR) [89]. It is known that inflammation can be triggered by oxidative stress caused by mitochondrial damage [90]. Our data demonstrated the accumulation of a mitochondrial abnormality and in turn, excessive oxidative stress in DM astrocytes. However, further studies are required to understand the molecular mechanisms through which excessive oxidative stress in astrocytes promotes inflammation. Although UA restores the mitophagy abnormality in DM astrocytes in vitro, the therapeutic effects of UA in DM optic nerve degeneration might not be limited to this. UA might also be beneficial through its direct effects on immune cells. UA could potentially offer therapeutic benefits in the treatment of common ophthalmic disorders of aging associated with mitochondrial dysfunction, including diabetic retinopathy, age-related macular degeneration, and glaucoma. Taken together, our findings indicate that mitochondrial health in astrocytes is essential to the homeostasis of the optic nerve and that UA has potential as a therapeutic agent for optic nerve degenerative diseases.

## Data Availability

All data generated or analyzed during this study are included in this published article (and its Supplementary Materials files).

## Supplementary Materials

The following supporting information can be downloaded at: https://www.mdpi.com/article/10.3390/cells12202496/s1, Figure S1: Inflammation is activated in DM optic nerve. Immunohistochemical analysis of the DM optic nerve showed the presence of CD4-positive T lymphocytes (arrows). Scale bar: 50 μm.; Figure S2: The mitochondrial membrane potential is reduced in DM astrocytes. Representative TMRM fluorescence intensity histogram from a flow cytometric analysis shows significantly lower fluorescence intensity in DM astrocytes corresponding to lower membrane potential. Data are mean ± SEM. n = 3, ** *p* < 0.01; Figure S3: mTORC1 is overactivated in DM optic nerves. (a,b) Optic nerves from all 4 genotypes were dissected and protein lysates were analyzed by immunoblotting. The representative immunoblotting images showed that the level of SQSTM1/p62, and the ratio of PS6:S6 are increased in the optic nerves of DM rats. Data are mean ± SEM from 3 independent experiments repeated in triplicate. * *p* < 0.05, ** *p* < 0.01; Figure S4: UA treatment induces autophagy in DM astrocytes. DM astrocytes were treated with UA (25 μM) for 24 h. In the last 3 h of culture, 50 nM BafA1 was added and the levels of autophagosome-positive LC3II isoform were analyzed by immunoblotting. A significant increase in the accumulation of autophagosomes positive for the LC3II isoform was observed in UA-treated cells relative to those untreated after BafA1 treatment. * *p* < 0.05.
