# Supplementary material for: Mitophagy in Astrocytes Is Required for the Health of Optic Nerve
Source: Cells. 2023 Oct 20;12(20):2496. doi: 10.3390/cells12202496 (PMC10605486; doi:10.3390/cells12202496)
Supplement: Supplementary file 1 [file cells-12-02496-s001.zip › cells-2614699-supplementary.pdf]

Supplementary Figures and Legends

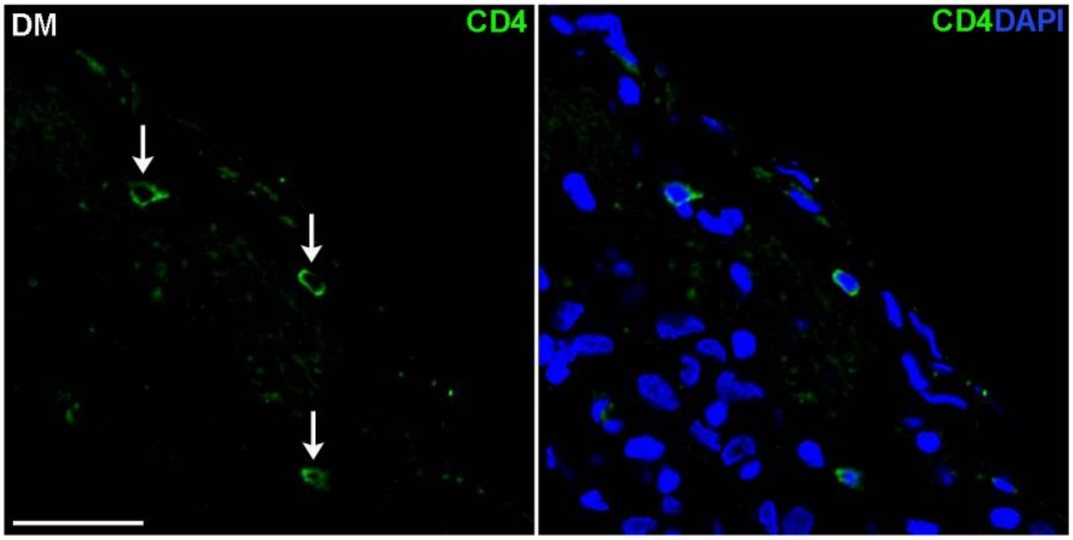

Supplementary Figure S1. Inflammation is activated in DM optic nerve. Immunohistochemical analysis of the DM optic nerve showed the presence of CD4-positive T lymphocytes (arrows). Scale bar: 50 μm.

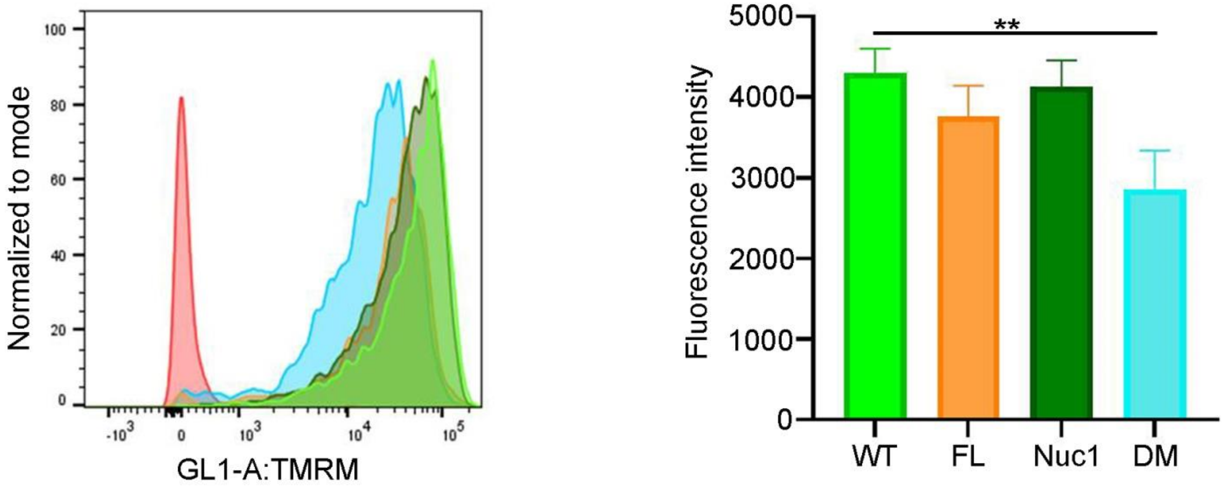

<u>Supplementary Figure S2</u>. The mitochondrial membrane potential is reduced in DM astrocytes. Representative TMRM fluorescence intensity histogram from a flow cytometric analysis shows significantly lower fluorescence intensity in DM astrocytes corresponding to lower membrane potential. Data are mean ± SEM. n = 3, ** $P < 0.01$.

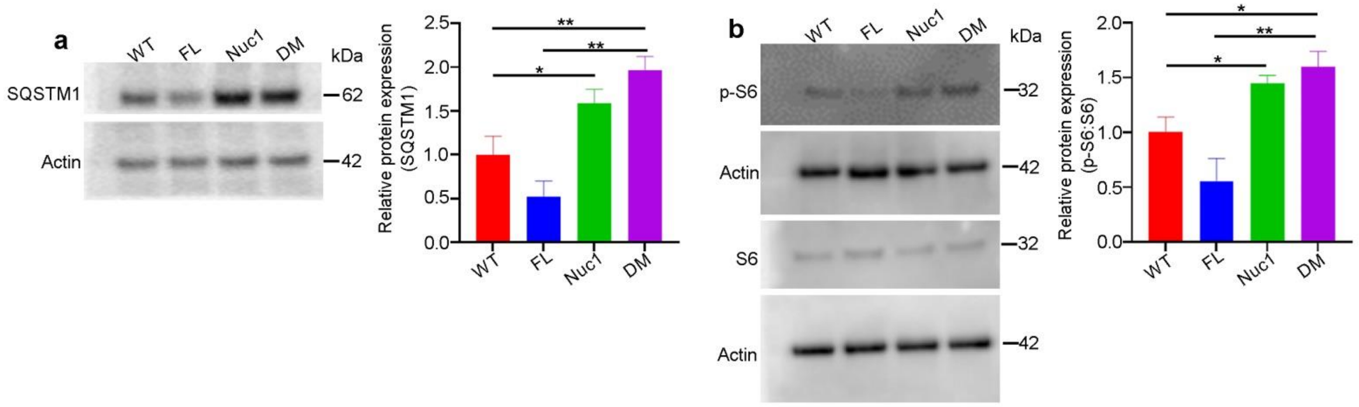

<u>Supplementary Figure S3</u>. mTORC1 is overactivated in DM optic nerves. (**a**,**b**) Optic nerves from all 4 genotypes were dissected and protein lysates were analyzed by immunoblotting. The representative immunoblotting images showed that the level of SQSTM1/p62, and the ratio of PS6:S6 are increased in the optic nerves of DM rats. Data are mean ± SEM from 3 independent experiments repeated in triplicate. * $P < 0.05$, ** $P < 0.01$.

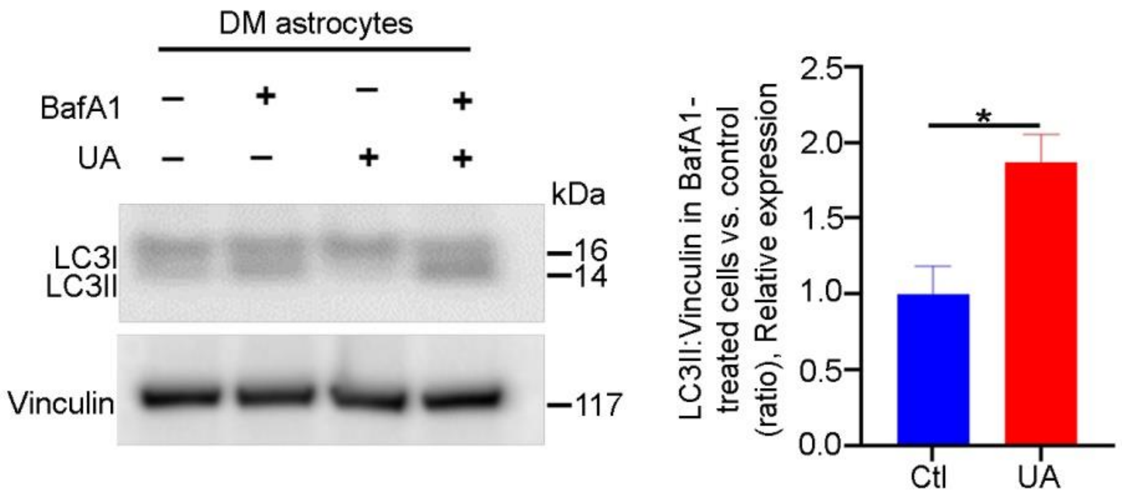

<u>Supplementary Figure S4</u>. UA treatment induces autophagy in DM astrocytes. DM astrocytes were treated with UA (25 μM) for 24 h. In the last 3 h of culture, 50 nM BafA1 was added and the levels of autophagosome-positive LC3II isoform were analyzed by immunoblotting. A significant increase in the accumulation of autophagosomes positive for the LC3II isoform was observed in UA-treated cells relative to those untreated after BafA1 treatment. * $P < 0.05$.
